# 

**Published:** 1891-11

**Authors:** 


					﻿Vol XI.
NOVEMBER, 1801.
No. 1.1;
THE
pOMEOPATHIC PHYSICIAN,
A Monthly Journal of Homoeopathic Materia Medica
and Clinical Medicine.
"He is a freeman whom truth makes free, and all are slaves beside."
"Seek the Truth: come whence it may, cost what it will."
EDITED AND PUBLISHED BY
WALTER M. JAMES, M. D.
PHILADELPHIA :
No. 1185 gPRTTCE 8TREET.
LO2STT3O 1ST :
ALFRED HEATH & CO., Sole Agents for Great Britain,
114 Ebury Street, Eaton Square.
Yearly Subscription, $2.SO. invariably in advance. Single numbers, 25 cts.
Entered at the Philadelphia Peet-Office aa Second- Claac Mall Matter.
				

